# Does Scarlet Fever Exist in the Lower Animals?

**Published:** 1884-10

**Authors:** Thomas Walley

**Affiliations:** Principal (Dick) Royal Veterinary College, Edinburgh


### Art. XXIII.—DOES SCARLET FEVER EXIST IN THE LOWER ANIMALS?

BY THOMAS WALLEY, M.R.C.V.S.,
Principal (Dick) Royal Veterinary College, Edinburgh.

At the close of the last day's proceedings of the meeting of the British National Veterinary Medical Association, I was requested by Dr. Stickler, who was present as a visitor, to state my opinion in reference to the existence of scarlet fever in the lower animals. As I was then, unfortunately, too much pressed for time to talk the matter over, I promised Dr. Stickler to write him at my earliest convenience. Since that meeting, I have had the opportunity of perusing in the April number of your Journal two articles on this subject—one by Dr. Peters, the other by Dr. Stickler; and after this perusal, it has occurred to me that the best plan I could adopt would be to state my views as publicly as these gentlemen have stated theirs.

In the first place, I may be allowed to remark that we in this country have neither the time nor the means to carry out pathological investigations as we would wish to do; consequently we are at a great disadvantage in these matters as compared with our transatlantic brethren—these disadvantages being added to by the heavy clog placed upon our movements by the vivisection act.

Secondly, I must remark that, *assuming* the transmissibility of scarlet fever of man to animals, or *vice versa*, to be a fact, and *assuming* the existence of this affection independently in animals, such transmissibility as such independent existence of the disease has never come under my immediate observation. But while saying this, I do not wish it to be understood that I disbelieve the statements of those who affirm to the contrary. I am, however, satisfied in my own mind that statements of a very sweeping character are often made which have no foundation in actual fact, and that a great tendency exists in the medical mind of the present day to accredit our domestic animals —particularly the cow—with the dangerous purport of being splendid media for the transmission of human zymotic diseases, or, according to some, of being actual generators of those diseases.

In connection with this subject, I may call attention to the twelfth annual report of the medical officer of the Local Government Board, 1882, in which a statement of Mr. Powers occurs, to the effect that in his opinion the cow may develop, immediately after parturition, a poison, which may have the power of inducing a scarlatinal form of disease in those persons who may partake of her milk at this period. If such were the case, we should hear more of scarlatinal attacks in those dairy districts in England in which the first milk—the beestings—is highly esteemed and largely used for the purpose of making custard (in Scotland this milk, unless given to the calf, is thrown away); and seeing that scarlatina, according to some authorities, is so easily propagated to the calf, we who have spent a large part of our lives in breeding and rearing districts, would most certainly have met with such cases if the cow is the progenitor of scarlatinal poison. The fact that milk is such a powerful absorber and retainer of all volatile matters, and that it is such a splendid pabulum for the nourishment of all forms of life, seems to me to be largely overlooked in inquiries into this and kindred subjects. The contamination of milk is, in my opinion, more often extrinsic than intrinsic.

A large part of Dr. Peters' article is devoted to purpura hæmorrhagica, and from the tenor of his remarks I gather that not only he, but other authorities whom he quotes, holds the opinion that this malady and scarlet fever are only different forms of the same affection. If in this I have interpreted him aright, I must at once dissent from such views. Scarlet fever is, to all intents and purposes, a specific eruptive fever, running a definite and prolonged course, marked by definite characteristics, and highly contagious. Can such be said of any form of purpura? I grant that in some outbreaks of scarlet fever purpuric symptoms are greatly exaggerated; but these cases are of a malignant type, and the same factor in the production of purpuric lesions is in existence as in ordinary cases of purpura, viz: an altered blood crasis.

Under what conditions do we see purpura in practice? Commonly in the horse after debilitating fevers, such as influenzas and strangles, and even common cold, particularly where the fever has been suppressed, the elimination of the fever poison and the products of histolysis not favored by appropriate medicines; or the administration of nutrient matters, to preserve the tissues from oxidation, has been neglected, or the patients have been placed under bad hygienic conditions, or have been supplied with impure water. Such is the history and origin of purpura in these cases; and it is a remarkable fact that those who pooh-pooh the nutrient and stimulant system of treatment in the continued fevers of the horse get the largest percentage of purpuric sequelæ.

The inhalation of contaminated air alone, it is stated, is sufficient to produce purpura. Such has not been my experience, unless the inhalation has been accompanied by some other conditions. Certainly the inhalation by cattle of the foul air in the holds of ships produces a malignant form of catarrhal fever, in which there is soreness of the throat, nasal catarrh, schneiderial, and even laryngeal ulceration, with systemic, serous, and in some cases hæmal effusions; but in the case of horses exposed for a long period to the influence of foul air in badly-ventilated stables, or in stables in which the drains have an open communication with sewers, the type of disease engendered has, in my experience, been a low form of relapsing or remitting fever, in which, while no purpuric symptoms have been developed during life, dropsies (ascites, etc.) have occurred as sequelæ, in which the blood has presented marked histological changes. That purpura may be due to defective or improper dieting, is also a well known fact, as is sometimes shown in sheep and cattle when feeding upon large quantities of succulent and highly-forced vegetable matters, such as grass, saintfoin, turnips, etc.; and in these cases the throat is frequently affected and the bowels relaxed, dysentery sometimes setting in prior to death. Impure water will, both in horses and cattle, induce febro-purpuric conditions; and of this I had a striking example some years ago in the case of a very valuable Clydesdale gelding, which, after a severe attack of catarrhal fever, presented all the symptoms of purpura hæmorrhagica, with *marked fever* and *marked throat complication*, and, as is often the case, marked metastasis of the purpuric lesions and periodic exacerbations of the symptoms. For some time this case puzzled me, more particularly as it did not yield to treatment; but at last I discovered that a water barrel to which the horse had free access, contained a large sediment of decomposing organic matter.

After the removal of this, and the administration of quinine and ergot of rye, the horse made a rapid and permanent recovery. In the dog and pig, purpura is not infrequently produced by the ingestion of putrefying fish or flesh; and we are all conversant with the fact that various forms of purpura can be produced, synthetically as it were, by the intra-venous injection of agents which will exercise a powerfully solvent effect in the colloids and cells of the blood, as, e. g., phosphorus, ammonia, sodium carbonate, biliary matters, or decomposing organic matter. Rheumatism, too, of a chronic type, is sometimes complicated with purpura; and I have seen several such cases in the horse.

Now, in all these forms of purpura, we have conditions in existence preëminently calculated to alter the normal relations existing between the different constituents of the blood, and to bring about marked nutritive changes in the walls of the blood vessels, thus leading either to serous or hæmal effusions, according as the disturbance is greater in the blood itself, or in the blood and the walls of the vessels together; but will any one assert that there is in any of these cases a single specific element?—or has any one ever seen them assume contagious characters? If so, it has not been my lot to see it. The argument that the contagious nature of purpura is proved by the fact that if the fluids from purpuric local lesions are injected under the skin of healthy animals, certain phenomena of a scarlatinal character are produced, is, in my opinion, worthless, for such phenomena can be produced by the injection of almost any organic fluid in a state of change.

While I have stated that it has not as yet been my lot to see distinct enzootics or epizootics of scarlet fever, I must acknowledge that I have occasionally seen, during an outbreak of influenza, the development of petechiæ on the schneiderial mucous membrane, accompanied by sore throat, local swellings, and tumefaction of the lymphatic glands about the throat; but such cases, I have always considered, are due to the altered state of the blood, and the symptoms disappear, under appropriate treatment, in the course of a few days. In horses fed on rye grass grown with nitrate of soda, it is no uncommon thing to have developed acute cases of inflammatory œdema of the limbs, with a marked tendency to metastatic translation to the intestines, the throat, or the lungs, and occasionally petechiæ on the visible mucous membranes, as of the nose or vagina.

In the horse, in the pig, and in the dog we have frequently cases of urticaria purpurans produced by the ingestion of improper food, or by exposure to chills after drinking large quantities of cold water, especially if the system is overheated at the time; and I am quite satisfied that in many instances these cases are mistaken for scarlatina, and described as such.

In concluding this article, allow me again to say that it has not been written with the object of cavilling, but merely for the purpose of recording my own opinion and experience in reference to a very important subject, and also in answer to Mr. Stickler's inquiry.

